# Do Premenopausal Women with Major Depression Have Low Bone Mineral Density? A 36-Month Prospective Study

**DOI:** 10.1371/journal.pone.0040894

**Published:** 2012-07-27

**Authors:** Giovanni Cizza, Sima Mistry, Vi T. Nguyen, Farideh Eskandari, Pedro Martinez, Sara Torvik, James C. Reynolds, Philip W. Gold, Ninet Sinai, Gyorgy Csako, for the POWER Study Group

**Affiliations:** 1 Section on Neuroendocrinology of Obesity, National Institutes of Diabetes and Digestive Kidney Diseases (NIDDK), National Institutes of Health, Bethesda, Maryland, United States of America; 2 Tulane University Internal Medicine-Pediatrics Residency Program, Tulane University School of Medicine, New Orleans, Louisiana, United States of America; 3 Behavioral Endocrinology Branch, National Institute of Mental Health, National Institutes of Health, Bethesda, Maryland, United States of America; 4 Clinical Center, National Institutes of Health, Bethesda, Maryland, United States of America; University of Tor Vergata, Italy

## Abstract

**Background:**

An inverse relationship between major depressive disorder (MDD) and bone mineral density (BMD) has been suggested, but prospective evaluation in premenopausal women is lacking.

**Methods:**

Participants of this prospective study were 21 to 45 year-old premenopausal women with MDD (n = 92) and healthy controls (n = 44). We measured BMD at the anteroposterior lumbar spine, femoral neck, total hip, mid-distal radius, trochanter, and Ward's triangle, as well as serum intact parathyroid hormone (iPTH), ionized calcium, plasma adrenocorticotropic hormone (ACTH), serum cortisol, and 24-hour urinary-free cortisol levels at 0, 6, 12, 24, and 36 months. 25-hydroxyvitamin D was measured at baseline.

**Results:**

At baseline, BMD tended to be lower in women with MDD compared to controls and BMD remained stable over time in both groups. At baseline, 6, 12, and 24 months intact PTH levels were significantly higher in women with MDD *vs.* controls. At baseline, ionized calcium and 25-hydroxyvitamin D levels were significantly lower in women with MDD compared to controls. At baseline and 12 months, bone-specific alkaline phosphatase, a marker of bone formation, was significantly higher in women with MDD *vs.* controls. Plasma ACTH was also higher in women with MDD at baseline and 6 months. Serum osteocalcin, urinary N-telopeptide, serum cortisol, and urinary free cortisol levels were not different between the two groups throughout the study.

**Conclusion:**

Women with MDD tended to have lower BMD than controls over time. Larger and longer studies are necessary to extend these observations with the possibility of prophylactic therapy for osteoporosis.

**Trial Registration:**

ClinicalTrials.gov NCT 00006180

## Introduction

Major Depressive Disorder (MDD) is a common condition affecting 98.7 million people globally [1] and nearly 35 million adults in the United States [2]. This chronic condition, characterized by depressed mood and/or anhedonia that interfere with activities of daily living, is a major cause of disability worldwide. By the year 2020, MDD will become second only to ischemic heart disease in the amount of disability experienced by sufferers of all ages according to the World Health Organization Global Burden of Disease Survey. The economic impact of depression is estimated in the tens of billions of dollars: depression cost employers over $40 billion dollars annually in lost productive work time [3]. MDD, once considered a disease only of the psyche, is now known to be associated with a number of medical conditions including cardiovascular disease [4]–[8], immune alterations [9]–[12], insulin resistance [13]–[16], diabetes mellitus [17]–[20], and obesity [21]–[24]. We and others have shown that depression is also associated with osteoporosis [25]–[42], yet depression is rarely listed as a risk factor for osteoporosis.

Unlike most physical illnesses observed in conjunction with MDD, osteoporosis is primarily asymptomatic and often remains undiagnosed until patients sustain pathologic fractures later in their lives. Due to the insidious presentation of osteoporosis, any concomitant mood change is unlikely to be reactive in nature. Although a few cross-sectional and cohort studies examining the relationship between depression and low bone mineral density (BMD) have been reported in pre- and post-menopausal women, there has been no prospective evaluation in premenopausal women [30]. Therefore, to investigate over time the association between BMD and depression in this population, we conducted a three-year prospective study by monitoring BMD over time in premenopausal women with MDD and healthy controls.

## Materials and Methods

### Participants

Participants of the *P*remenopausal, *O*steoporosis, *W*omen, Alendronat*e*, Dep*r*ession (POWER) study were 21- to 45-year-old premenopausal women with current or recent MDD (n = 92) and healthy control women (n = 44). Recruitment took place from July 1, 2001, to February 28, 2003, in the Washington, DC, metropolitan area by newspaper and radio. Internet and flyer advertisement [27]. Women with MDD were enrolled if they met the Diagnostic and Statistical Manual of Mental Disorders, 4th. Edition (*DSM*-IV) criteria for MDD and experienced a depressive episode in the preceding three years; a limit chosen to minimize recall bias associated with more remote depressive episodes.

Exclusion criteria for women with MDD included eating disorders, bipolar disorders, schizophrenia, schizoaffective disorder, and suicidal risk. Patients with anxiety disorders or a history of drug or alcohol dependence in remission for at least five years were eligible. Subjects were allowed to continue their antidepressant treatments under the care of their physician. Hyperthyroidism, vitamin D deficiency and other conditions and treatments that affect bone turnover were additional exclusion criteria. Exclusion criteria for controls were a T-score equal to or lower than −1.5 at the anterior-posterior (AP) lumbar spine, femoral neck or total hip and a history of any DSM-IV diagnosis apart from prior alcohol abuse. Pregnancy and menopause were additional exclusion criteria [27].

**Figure 1:**
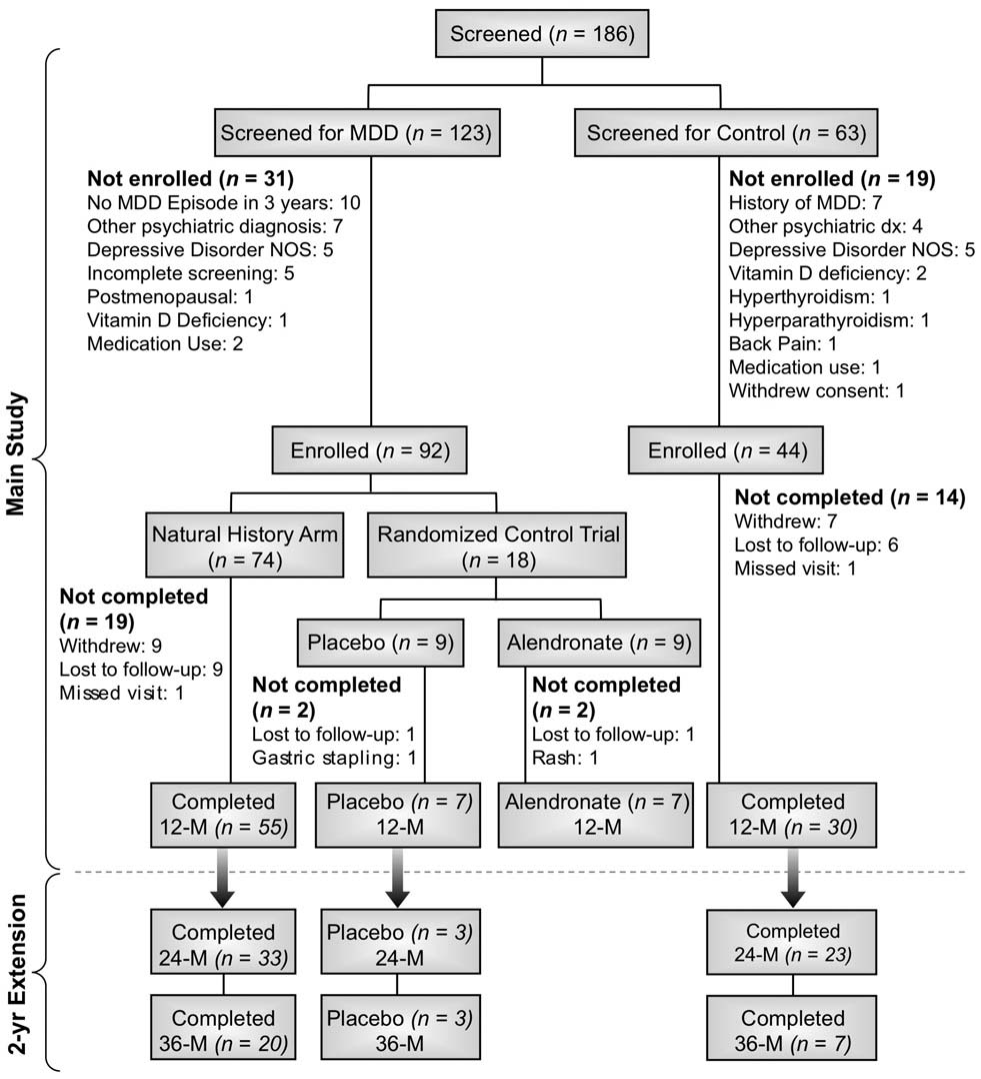
Study flow diagram. Note: The number of exclusions does not match the number of people as some participants were found to have more than one exclusionary criterion.

The health status of each subject was evaluated by medical history and physical examination. Screening electrocardiogram, serum pregnancy test, complete metabolic panel, complete blood count, 25-hydroxyvitamin D, intact parathyroid hormone (iPTH), thyrotropin, and free thyroxine and urine toxicology screen were obtained. Figure 1 describes the number of individuals screened and the reasons for exclusion. Of note, none of the control subject screened had a T-score equal to or lower than −1.5 at the anterior-posterior (AP) lumbar spine, femoral neck or total hip. The National Institute of Mental Health's Institutional Review Board and the Scientific Review Board approved the original 12 month study and its subsequent extension to 36 month. In addition, all subjects provided written informed consent. The trial was registered in ClinicalTrials.gov, NCT 00006180.

### Study design

The POWER Study was designed as a 12-month investigation consisting of: 1) a longitudinal follow-up comparison of BMD in women with MDD and controls (Natural History Arm) and; 2) a randomized, double-blind, placebo-controlled, 12- month trial of alendronate in women with MDD with moderate osteopenia (Clinical Trial Arm). Further details on study design have been previously reported [27]. In the Clinical Trial Arm women with MDD, who at baseline had a T-score equal to or lower than −1.5 at the anterior-posterior (AP) lumbar spine, femoral neck or total hip (n = 14), were randomized to 70 mg of alendronate (n = 7) or matching placebo tablets (n = 7) orally once a week (Merck & Co., Inc., Rahway, NJ). In addition, both groups in the Clinical Trial Arm received 500 mg daily of elemental calcium and 400 IU of vitamin D.

We subsequently extended the study to a total of 36 months (Figure 1). At the end of the 12-month main study, subjects from the Natural History Arm and those subjects in the Clinical Trial Arm that were randomized to placebo were offered continued participation in an additional 24-month study extension to assess bone mineral density and biochemical markers of bone turnover at yearly intervals (“Extended Natural History Arm”).

### Procedures

#### BMD, biochemical markers of bone turnover, and hormonal measurements

BMD was measured by dual-energy x-ray absorptiometry (DXA QDR 4500 machine; Hologic Inc., Bedford, MA) at the following sites: anteroposterior lumbar (L1–L4) spine, total hip, femoral neck, trochanter, Ward's triangle, and mid-distal radius. The coefficient of variation was <0.4% at each site. DXAs were analyzed by the study radiologist, J.R., blinded to group allocation. Two markers of bone formation, serum bone-specific alkaline phosphatase and 8:00 AM osteocalcin, and a marker of bone resorption, urinary N-telopeptide, were assessed. 8:00 AM plasma adrenocorticotropic hormone (ACTH), 8:00 AM serum cortisol, 24-hour urinary-free cortisol, serum iPTH, plasma 25-hydroxyvitamin D and ionized serum calcium were also obtained. These measurements were obtained at 0, 6, 12, 24 and 36 month. 25-Hydroxyvitamin D plasma levels were only measured at baseline.

#### Psychiatric evaluation

We administered the structured clinical interview (SCID) for DSM-IV Axis I disorders and enrolled subjects if they met DSM-IV criteria for MDD and had an episode of major depression in the past three years (SCID). The Hamilton Depression Scale (HAM-D) and Hamilton Anxiety Scales (HAM-A) were used to determine the severity of depression and anxiety in study participants at baseline, 12, 24 and 36 months.

#### Life style risk factors for osteoporosis

Calcium from food and supplements, caffeine, and alcohol intake were assessed using a food frequency questionnaire. A nutritionist informed the subjects of their calcium intake and recommended to consume 1000 mg/day of calcium [27]. Cigarette smoking history and oral contraceptive use were also recorded. The Cooper test (12-minute walk/run test) was administered as an indirect index of physical fitness, and was measured in meters traversed within 12-minutes on a standardized treadmill [27].

#### Anthropometric measurements

As previously described, height was measured to the nearest 0.1 cm using a stadiometer and weight to the nearest 0.1 kg using a digital scale [27]. BMI was calculated as kg/m^2^.

### Statistical analyses

Data are reported as mean (SD) or by frequencies and percents, unless otherwise indicated. Differences between groups (MDD and control subjects, or between the clinical trial treatment arms) were tested by the *t*-test (or non-parametric parallel, as necessary) and Fisher exact test, as appropriate. Paired data between time intervals utilized the paired *t*-test (or non-parametric parallel, as necessary) for continuous variables or McNemar test for categorical ones. The relationship between depression and BMD was assessed by analysis of covariance, adjusting for BMI. In women with MDD, the association of BMD with clinical parameters of depression and anxiety was assessed by linear regression. Repeated-measures analysis of variance (ANOVA) using mixed modeling was used to compare changes in BMD over time, and was adjusted for BMI. All analyses were done using SAS v9.2 (SAS Institute Inc, Cary, NC), and all tests were 2-sided with a significance level of 0.05.

## Results

### Clinical characteristics of study participants over time

Participant retention rate over the course of the main study was not significantly different between groups (women with MDD 78%; control women 68%; P = 0.211). The subjects who elected to enroll in the study extension did not differ in demographic characteristics from those who did not (data not shown). Of note, only 2 of the 18 women with depression participating in the Clinical Trial Arm were lost to follow-up.

Our sample was composed of mostly white, college-educated women (Table 1). Demographic characteristics were not different between women with MDD and controls at any of the study follow-up phases, but women with MDD tended to have a higher BMI and tended to be less often married than control women. Women with MDD reached menarche one year earlier than controls but had a similar number of pregnancies and a similar current use of OCP than controls. Alcohol use was less common in women with MDD.

**Table 1:** Baseline demographic, lifestyle and clinical characteristics of study participants included at various study follow-up phases.*

	BASELINE			12-MONTH STUDY			24- MONTH EXTENSION STUDY		
Characteristics	MDD Women (*n* = 92)	Control Women (*n* = 44)	P	MDD Women (*n* = 72)	Control Women (*n* = 30)	P	MDD Women (*n* = 36)	Control Women (*n* = 23)	P
Age, *y*	36.0 (6.9)	35.3 (6.9)	0.50	36.0 (6.9)	36.0 (6.8)	0.91	38.2 (6.3)	36.6 (7.1)	0.41
BMI, *kgm^2^*	26.4 (6.2)	24.1 (3.7)	0.10	25.8 (5.6)	23.8 (3.4)	0.23	26.3 (5.1)	23.6 (2.9)	0.06
Race (White), %	87	86	1.00	88	90	1.00	94	96	1.00
Education, *y*	16.4 (2.1)	16.3 (2.1)	0.66	16.7 (2.0)	16.7 (2.1)	0.94	16.5 (1.8)	16.4 (1.9)	0.85
Married, %	36	48	0.26	35	53	0.12	39	61	0.12
Age Menarche, *y*	12.5 (1.6)	13.0 (1.6)	0.11	12.6 (1.5)	13.1 (1.6)	0.17	12.7 (1.3)	13.0 (1.3)	0.71
No. Pregnancies	1.2 (1.6)	1.2 (1.3)	0.59	1.0 (1.5)	1.3 (1.5)	0.32	1.3 (1.7)	1.4 (1.5)	0.81
Current OCP, %	32	34	0.85	32	30	1.00	19	22	1.00
Alcohol Use, %	70	98	**<0.001**	81	97	0.06	89	100	0.15
History of smoking, %	40	34	0.58	41	27	0.26	44	22	0.10
Calcium intake, *mg/d* ‡	1396 (663)	1385 (734)	0.71	1356 (627)	1461 (810)	0.78	1392 (618)	1550 (856)	0.61
Caffeine intake, *mg/d*	215 (261)	217 (164)	0.43	193 (251)	219 (164)	0.27	208 (282)	202 (155)	0.53
Cooper test§, *m/12 min*	1316 (385)	1400 (254)	0.12	1328 (386)	1398 (273)	0.23	1236 (356)	1402 (247)	**0.03**

Abbreviations: BMI, body mass index (calculated as weight in kilograms divided by height in meters squared); MDD, major depressive disorder; OCP, oral contraceptive pill.

* All values expressed as mean (SD), unless otherwise specified.

‡ Calcium intake calculated from dietary and supplement sources.

§ Index of physical fitness, meters covered in 12-minut

**Figure 2:**
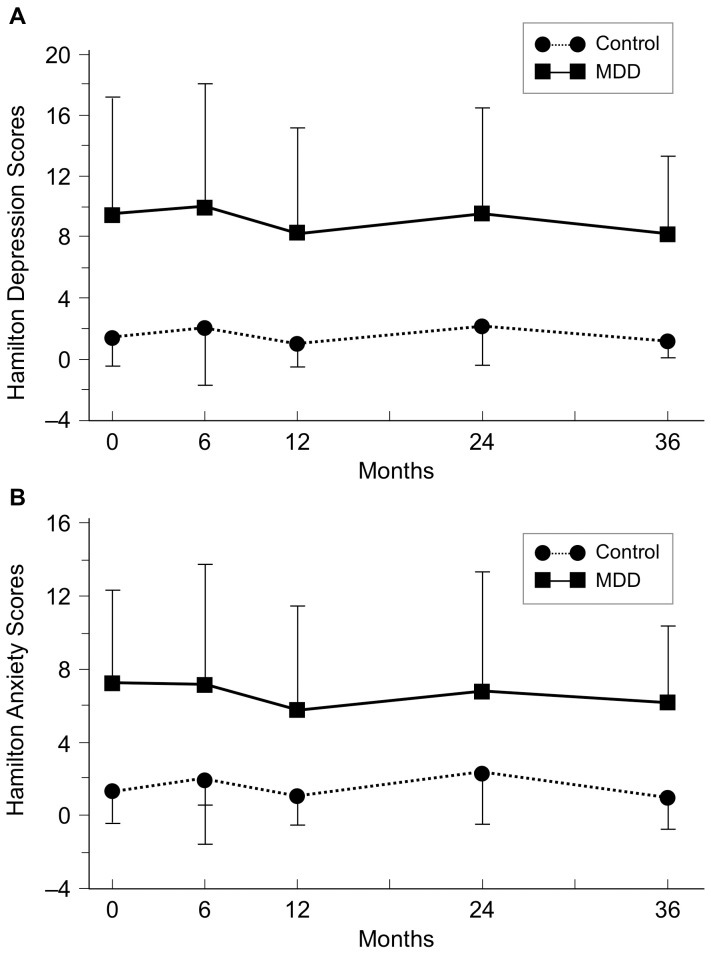
Hamilton depression (upper panel) and anxiety (lower panel) scores in women with MDD and control women over time. Both depression and anxiety scores were relatively low and remained stable over time in women with MDD. As expected, scores for depression and anxiety were much higher in women with MDD *vs.* control women.

At baseline, only one-fifth of women with MDD (17/92) had current depression defined as a major episode within the last month. This sample of women with MDD however, had a considerable lifetime burden of depression, as indicated by cumulative history (68.6±77.9 months) and total number of depressive episodes (5.9±11.4). Age of onset was in the late teens (19±9 years old). Approximately half (52%) of the women had other *DSM-IV* axis I diagnoses, mostly anxiety disorders (not shown). Finally, 81 out of 92 women were taking antidepressants, 70% a selective serotonin reuptake inhibitor (SSRI), and 30% another antidepressant. Hamilton anxiety and depression scores were relatively low in women with depression consistent with their remission state, and remained stable over time (Figure 2).

### BMD over time in the Extended Natural History Arm

At baseline, BMD was between 2% and 3% lower in women with MDD at the main skeletal sites, however, these differences did not reach statistical significance (Table 2). The prevalence of osteopenia appeared consistently greater in women with MDD compared to control women at the total hip and femoral neck. Over 36 months, there was no decline in BMD in either group.

**Table 2:** BMD values adjusted by BMI in women with depression versus control women at each main skeletal site.^*^

	BASELINE			6-MONTH			12-MONTH			24-MONTH			36-MONTH		
Site	Control	MDD	P	Control	MDD	P	Control	MDD	P	Control	MDD	P	Control	MDD	P
**AP Spine**															
**Density, g/cm^2^**	1.053 (0.016)	1.021 (0.011)	0.10	1.072 (0.015)	1.040 (0.011)	0.10	1.067 (0.017)	1.041 (0.012)	0.22	1.063 (0.022)	1.051 (0.018)	0.69	1.103 (0.042)	1.052 (0.023)	0.30
**T-score**	−0.014 (0.143)	−0.266 (0.098)	0.44	0.132 (0.137)	−0.090 (0.103)	0.20	0.148 (0.154)	−0.067 (0.107)	0.26	0.146 (0.194)	0.068 (0.153)	0.76	0.482 (0.369)	0.085 (0.199)	0.36
**Percent Osteopenia, %**	15.9	18.9	0.81	14.7	15.0	1.00	16.7	16.4	1.00	17.4	11.1	0.70	00	17.4	0.55
**Total hip**															
**Density, g/cm^2^**	0.986 (0.0163)	0.956 (0.011)	0.13	0.991 (0.019)	0.970 (0.014)	0.38	0.982 (0.020)	0.965 (0.014)	0.48	0.969 (0.020)	0.968 (0.016)	0.97	0.998 (0.037)	0.965 (0.020)	0.45
**T-score**	0.294 (0.130)	0.058 (0.089)	0.14	0.306 (0.146)	0.209 (0.109)	0.60	0.303 (0.158)	0.157 (0.110)	0.45	0.203 (0.166)	0.193 (0.130)	0.96	0.400 (0.312)	0.157 (0.168)	0.51
**Percent Osteopenia, %**	2.3	15.2	**0.04**	2.9	11.7	0.25	0	14.8	**0.03**	4.4	11.1	0.64	14.3	13.0	1.005
**Femoral neck**															
**Density, g/cm^2^**	0.878 (0.016)	0.843 (0.011)	0.39	0.878 (0.0178)	0.852 (0.013)	0.25	0.858 (0.019)	0.850 (0.013)	0.72	0.838 (0.021)	0.845 (0.017)	0.82	0.857 (0.041)	0.831 (0.022)	0.59
**T score**	0.179 (0.135)	−0.116 (0.093)	0.37	0.174 (0.154)	−0.025 (0.115)	0.31	0.046 (0.168)	−0.033 (0.117)	0.71	−0.113 (0.188)	−0.014 (0.925)	0.69	−0.003 (0.360)	−0.210 (0.193)	0.62
**Percent Osteopenia, %**	4.6	17.4	0.06	8.8	15.0	0.53	13.3	13.1	1.00	30.4	11.1	0.09	28.6	13.0	0.57
**Radius**															
**Density, g/cm^2^**	0.712 (0.008)	0.695 (0.005)	0.13	0.711 (0.008)	0.670 (0.006)	0.19	0.710 (0.009)	0.694 (0.006)	0.14	0.715 (0.011)	0.698 (0.008)	0.24	0.719 (0.020)	0.706 (0.011)	0.57
**T score**	0.344 (0.124)	−0.003 (0.085)	0.053	0.291 (0.134)	0.115 (0.100)	0.30	0.236 (0.143)	0.002 (0.098)	0.18	0.343 (0.175)	0.064 (0.137)	0.23	0.398 (0.329)	0.195 (0.177)	0.60
**Percent Osteopenia, %**	9.1	10.9	1.00	8.8	6.7	0.70	6.9	13.1	0.49	8.7	11.1	1.00	0	8.7	1.00

### Biochemical markers of bone turnover and hormones of hypothalamic-pituitary-adrenal (HPA) axis in the Extended Natural History Arm

Intact PTH levels were significantly higher in women with MDD *vs.* control women and generally remained higher up to 36 months (Table 3). Ionized calcium was lower in women with MDD at baseline and this difference was maintained across study duration. Vitamin D levels at baseline were significantly lower in women with MDD. Bone specific alkaline phosphatase, a marker of bone formation, was significantly higher in women with MDD at baseline and remained higher in this group across study duration, although only statistically significantly different at 12 months. Another marker of bone formation, serum osteocalcin, was not different between groups and neither was urinary N-telopeptide, a marker of bone resorption. The 8 am plasma ACTH was higher in women with MDD at baseline and 6 months only. The 8 am serum cortisol and urinary free cortisol levels were not different between groups at any time point.

**Table 3:** Calcium metabolism, bone turnover markers, and hormonal measurements in women with depression compared to control women.*

	BASELINE			6-MONTH			12-MONTH			24-MONTH			36-MONTHS		
Variable	Control	MDD	P	Control	MDD	P	Control	MDD	P	Control	MDD	P	Control	MDD	P
**iPTH, pg/mL**	37.00 (16.67)	43.84 (18.91)	**0.04**	31.85 (11.72)	41.71 (18.40)	**<0.01**	24.45 (5.704)	38.80 (18.51)	**<0.001**	25.35 (9.369)	31.28 (12.56)	0.07	26.26 (6.509)	37.64 (13.74)	**0.04**
**Ionized calcium, mmol/L**	1.261 (0.04139	1.242 (0.04755)	**0.03**	1.256 (0.03606)	1.242 (0.04800)	0.16	1.254 (0.03118)	1.262 (0.05033)	0.43	1.267 (0.02739)	1.265 (0.04738)	0.91	1.271 (0.03934)	1.272 (0.04695)	0.96
**25-Hydroxy-vitamin D, ng/mL** ‡	34.20 (2.267)	27.57 (1.112)	**<0.01**	N/A	N/A	N/A	N/A	N/A	N/A	N/A	N/A	N/A	N/A	N/A	N/A
**Bone-specific alkaline phosphatase, µg/L**	8.567 (2.201)	9.710 (2.726)	**0.03**	8.852 (2.099)	9.901 (3.054)	0.08	9.231 (2.526)	10.63 (3.190)	**0.04**	9.640 (2.395)	10.72 (3.716)	0.24	8.314 (1.878)	10.70 (3.623)	0.10
**8am Osteocalcin, ng/mL**	4.383 (1.822)	4.665 (3.593)	0.66	4.377 (1.222)	3.924 (1.863)	0.22	4.597 (1.379)	4.447 (1.620)	0.66	4.280 (1.084)	4.250 (2.175)	0.95	3.357 (1.391)	5.297 (3.653)	0.18
**Urinary N-telopeptide, nmol/mmol of creatinine**	24.08 (10.34)	21.18 (7.849)	0.09	23.06 (8.534)	24.99 (13.27)	0.46	24.04 (12.13)	23.11 (8.866)	0.68	20.19 (8.118)	23.88 (10.51)	0.21	17.00 (6.261)	26.04 (13.15)	0.11
**8am plasma ACTH pg/mL**	23.54 (8.410)	33.91 (27.30)	**0.02**	20.57 (7.365)	27.01 (16.05)	**0.03**	19.72 (7.005)	23.55 (13.84)	0.16	13.46 (6.291)	19.38 (14.25)	0.10	14.27 (6.434)	17.45 (8.912)	0.38
**8am serum cortisol, µg/dL**	19.52 (8.498)	20.36 (6.400)	0.54	18.37 (7.869)	18.33 (7.549)	0.98	17.40 (9.328)	17.23 (7.163)	0.93	11.85 (6.729)	14.50 (8.453)	0.22	10.60 (2.871)	13.08 (7.360)	0.39
**Urinary-free cortisol, µg/24 h**	62.19 (22.58)	57.57 (26.91)	0.35	44.95 (21.96)	49.47 (23.89)	0.38	50.04 (22.71)	47.13 (22.72)	0.58	43.82 (19.91)	41.69 (18.15)	0.72	52.33 (11.02)	42.30 (18.17)	0.36

* All values expressed as mean (SD), unless otherwise specified.

‡ 25-Hydroxyvitamin D, ng/mL levels were only obtained at baseline.

Sample size indicated in Table 1.

### Relationship between plasma cortisol and indices of clinical severity of depression and anxiety and BMD over time in the Extended Natural History Arm

There was no relationship between current depression, current treatment or current SSRI treatment *vs.* BMD and biochemical markers of bone turnover. Surprisingly, in women with MDD, both depression and anxiety scores were slightly positively related with BMD at the AP spine (BMD values after adjustment for BMI versus depression and anxiety scores, respectively: r = 0.173; p = 0.005; r = 0.136; p = 0.029) and trochanter (data not shown).

### BMD over time in the Clinical Trial Arm

**Figure 3:**
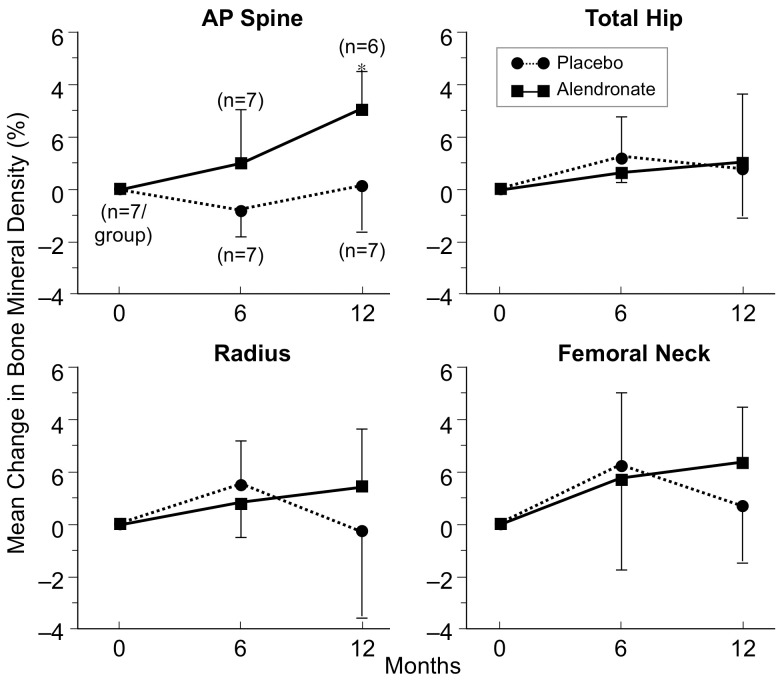
Bone mineral density measurements in women with MDD and moderate osteopenia or osteoporosis randomized to alendronate *vs.* placebo. Over 12 months, the Alendronate group showed a significant increase in BMD at the lumbar spine (P = 0.003), and there was a trend for increased BMD at the femoral neck (P = 0.06). No changes over time were observed in the Placebo group.

Fourteen out of 92 of women with MDD (20%) and none of the controls exhibited a T-score lower than −1.5 in at least one skeletal site. Thirteen of these women with MDD participated in the Clinical Trial Arm of the study (Figure 3). Patients in the placebo group compared to those randomized to alendronate had similar characteristics. Treatment with alendronate significantly increased BMD at the lumbar spine (0.8525±0.0312 g/cm^2^ *vs.* 0.8792±0.0379 g/cm^2^, P = 0.003; C.I., 0.01 to 0.04, baseline *vs.* 12-month) and tended to increase BMD at the femoral neck (0.7423±0.0735 *vs.* 0.7588±0.0709 g/cm^2^, P = 0.06). No changes over time were observed in the placebo group. Alendronate treatment decreased osteocalcin concentration (4.2±1.6 *vs.* 2.1±1.1 ng/ml, P = 0.04; C.I., −4.0 to −0.2, baseline *vs.* 12-month), but did not significantly affect bone-specific alkaline phosphatase or urinary N-telopeptide. There were no changes in biochemical markers in the placebo group.

## Discussion

Osteoporosis is a significant cause of morbidity and mortality in the US and costs approximately $17 billion dollars annually [43]. It results in over two million fractures annually in the US, 71% of which occur in women [43]. Therefore, investigation and identification of risk factors are of great importance. There have been several studies with conflicting findings regarding the possible influence of depression on BMD [25]–[42]; most of these studies were retrospective analyses conducted in post-menopausal women. In this study, we followed a group of premenopausal women with MDD and healthy controls prospectively and measured their BMD at regular intervals. Our finding that neither group exhibited a substantial change in BMD at any skeletal site over time may not be surprising since BMD has been reported to remain relatively stable in healthy premenopausal women [44]–[47]. The observation that women with MDD maintained their BMD throughout the study is reassuring as it implies that little, if any, bone loss was associated with MDD in this age range and time span. It should be however noted that these subjects were aware that they were participating in a clinical experiment, thus we cannot exclude a non specific “Hawthorne effect”. In this particular case, regular encounters with the research team may have positively influenced their mood and induced improvements in life style conditions. Our observation allows for considering prophylactic treatment of these women to prevent osteoporosis after menopause, when their risk is magnified. Women reach peak bone mass by their third decade [48], [49] and BMD remains relatively stable until menopause where women begin to lose up to 1–2% of the BMD annually [44]–[47].

Alterations in the HPA axis are significant findings in biological psychiatry [50]. Several studies have investigated the possible pathophysiology of osteoporosis in psychiatric patients and have hypothesized a link between depression and low BMD [28]. Elevated ACTH and cortisol levels, and enhanced cortisol responsiveness have been demonstrated in depressed individuals [25], [32], [41], [51]–[53]. Similar to the bone loss observed in Cushing syndrome as a result of hypercortisolemia, women with depression could thus have decreased BMD, albeit not as pronounced as in Cushing syndrome. We found that ACTH levels and bone-specific alkaline phosphatase levels tended to be elevated in women with depression compared to controls. Serum and urinary free cortisol, osteocalcin, and urinary N-telopeptide levels were not different between participants with depression and controls throughout the study.

In an ancillary investigation, we reported that women with depression had a greater prevalence of *Bcl1* polymorphism, which is associated with glucocorticoid hypersensitivity [54]–[56]. Therefore, women with MDD may also have a greater HPA activity at tissue level. The cortisol plasma levels were not elevated in our study of women with MDD, but hyperactivity of the HPA axis is not always accompanied by hypercortisolism [57]. Alterations in the HPA axis tend to occur during acute depressive states and normalize after treatment [51], [53]. Given that the majority of the subjects with depression were being pharmacologically treated throughout the duration of the study as previously reported [27], increases in cortisol levels may not have occurred, as these patients were likely to be in clinical remission. As we have recently reported in greater detail in a related manuscript [58], approximately half of the sample was comprised of women with melancholic depression, and the remaining subjects suffered either from undifferentiated or atypical depression. Women with atypical features of depression had higher ACTH levels during the night and women with undifferentiated depression had a significantly higher prevalence of low BMD at the femoral neck than controls. Thus, the clinical subtype of depression may influence bone and endocrine features, among other parameters.

In the women with MDD and moderate osteopenia or osteoporosis, weekly alendronate was effective in increasing BMD. This is the first pharmacotherapeutic study of osteoporosis in younger women with MDD and one of the few controlled studies of alendronate treatment in premenopausal women [59]–[61]. Of note, in this arm the drop-out rate was only 10%, much smaller than in the overall cohort. In future research, it would be interesting to identify the predictors of drop-out rate in studies of women with depression. It is possible that the women participating in the randomized controlled arm of this trial may have been more motivated to remain in the study than the women with depression in the natural history arm and the normal controls, possibly because of the therapeutic advantages of the drug being received. As estrogens are not a treatment option in this population, our study supports the possible use of alendronate in this population. Recently the use of selective serotonin reuptake inhibitors has been linked to an increased risk of fractures and bone loss [62]. As reported [27], in our study the use of selective serotonin reuptake inhibitors was not associated with low BMD.

Vitamin D levels were lower in women with MDD than controls. Consistent with decreased vitamin D levels, women with MDD had significantly higher iPTH and ionized calcium levels, highly suggestive of secondary hyperparathyroidism. PTH levels remained elevated in women with MDD compared to controls. While elevated PTH levels have been demonstrated in depressed elderly women and young men [36], [63], [64], to the best of our knowledge, this is the first time elevated PTH plasma levels are observed in premenopausal women with MDD. Future studies should be conducted to evaluate the pathogenetic role of secondary hyperparathyroidism in subjects with depression.

### Study limitations and merits

The small sample size, together with a drop-out rate of approximately 30% in the first year, may have reduced our ability to detect some associations. Vitamin D levels were only measured at baseline. Furthermore, since there is little bone loss in this age range [44]–[47], the duration of the study and the age of the participants may have limited our ability to identify a subtle decrease in BMD. Our results may have failed to detect significant changes in biochemical markers and hormones in patients who were in remission since abnormalities in many of these parameters might only be apparent during acute disease states [51], [53]. Our sample was well characterized and homogeneous, and the length of follow-up was longer than most studies of this kind.

### Conclusion

Premenopausal women with MDD had lower BMD than controls over a sustained period of time. Larger and longer studies are needed to confirm and extend these observations. The effects of antidepressants and other psychotropic medications on bone mass *per se* should be assessed. The reversibility of bone loss due to successful behavioral or pharmacologic interventions or to spontaneous resolution of depression should be considered. Lastly, studies examining the role of genetics leading to enhanced susceptibility to reductions in BMD need to be conducted.
