# Correction: Do Premenopausal Women with Major Depression Have Low Bone Mineral Density? A 36-Month Prospective Study

**DOI:** 10.1371/annotation/193f2b29-b8b0-41e8-808a-290df5577a53

**Published:** 2012-10-15

**Authors:** Giovanni Cizza, Sima Mistry, Vi T. Nguyen, Farideh Eskandari, Pedro Martinez, Sara Torvik, James C. Reynolds, Philip W. Gold, Ninet Sinaii, Gyorgy Csako, for the POWER Study Group

The ninth author's name was spelled incorrectly. The correct name is: Ninet Sinaii.

